# 

**Published:** 1893-04-01

**Authors:** 


### CONTENTS.

Experiment and Experience - - - - - - - - 1

Around the Hospitals - - - - - - - - - 2

Medical History from the Earliest Times. By E. T. WITHINGTON, M.A., M.B Oxon. XLVIII.—Paracelsus (*continued*) 3

Contemporary Theory and Research - - - - - 4

Diet in Disease. By Mrs. ERNEST HART. XVIII.—Indigestion - 5

Gleanings in Science and Medicine - - - - - - 6

Annotations.—Is Massage a Swindle?—Dr. Klein and Cholera Inoculation—The Presidency of the R. C. P. - - - - - 7

Notes and News - - - - - - - - - - 8

A Study in Modern Socialism.—"The Social Horizon" - 13

The Hospital Clinic—SUSSEX COUNTY HOSPITAL.—Treatment of Simple Fracture of the Femur - - - - - - - 9

LIVERPOOL ROYAL INFIRMARY.—The Treatment of Pleural Effusion - - - - - - - - - - 11

THE PRACTITIONERS' BOOKSHELF.—Gynæcology—Inorganic Chemistry - - - - - - - - - - 12

NEW DRUGS, APPLIANCES, AND THINGS MEDICAL.—Milled Toilet Soaps - - - - - - - - - - - 12

Construction Notes - - - - - - - - - - 16

Scraps and Gleanings - - - - - - - - - 16

Book World of Medicine and Science - - - - -

### EXTRA SUPPLEMENT.—THE NURSING MIRROR.

News from the Nursing World.—A Royal Guest—Royal Patronesses—Gratitude—Valued Workers—"A Plea for Sick Paupers"—Free Libraries—Only Four Minutes—Liberality at Burton-on-Trent—Ingenious Disinfection—Free Conveyance Bereavement and Bitterness—Faithful Service—Short Items—The Disabled Nurse—Ordinary Nurses - - - - - i
Management of Consumption. VIII.—Occupation for the Consumptive - - - - - - - - - - - iii
Novelties for Nurses - - - - - - - - - iii
Appointments - - - - - - - - - - - iii
Nursing at Natal—A Private Nurse's Opinion - - - - iv
Attainable Perfection - - - - - - - - - v
Notes and Queries - - - - - - - - - - v
Thoughts About Pictures for Holy Week - - - - vi
Everybody's Opinion.—Convalescent Home—Borrowed Without Acknowledgment—Nurses for Norfolk - - - - - vii
For Reading to the Sick.—Christ's Loved Ones - - - vii
An Infallible Cough Mixture. A House Surgeon's Story. II. viii
Wants and Workers - - - - - - - - - viii
The Book World for Women and Nurses - - - - ix

